# Comprehensive safety assessment and therapeutic potential of *Pediococcus acidilactici* NMCC-B in attenuating arthritis progression

**DOI:** 10.1371/journal.pone.0324060

**Published:** 2025-05-22

**Authors:** Tayyaba Zulfiqar, Muhammad Khalid Tipu, Muhammad Tariq Khan, Amr S. Abouzied, Bassam S. M. Al Kazman, Saud O. Alshammari, Qamar A. Alshammari, Abdulkarim Alshammari, Muhammad Nasir Hayat Malik, Amir Ali, Muhammad Usama Mazhar, Hafsa Jabeen

**Affiliations:** 1 Department of Pharmacy, Faculty of Biological Sciences, Quaid-i-Azam University, Islamabad, Pakistan; 2 Department of Pharmaceutical Chemistry, College of Pharmacy, University of Hail, Hail, Saudi Arabia; 3 Department of Pharmacognosy, College of Pharmacy, Najran University, Najran, Saudi Arabia; 4 Department of Pharmacognosy and Alternative Medicine, College of Pharmacy, Northern Border University, Rafha, Saudi Arabia; 5 Department of Pharmacology and Toxicology, College of Pharmacy, Northern Border University, Rafha, Saudi Arabia; 6 Department of Pharmacy Practice, College of Pharmacy, Northern Border University, Rafha, Saudi Arabia; 7 Faculty of Pharmacy, The University of Lahore, Lahore, Pakistan; 8 Faculty of Pharmacy, Capital University of Science and Technology, Islamabad, Pakistan.; University of Rijeka Faculty of Medicine: Sveuciliste u Rijeci Medicinski fakultet, CROATIA

## Abstract

Dysbiosis of gut microbiota and loss of gut-barrier integrity contribute to the development and severity of rheumatoid arthritis (RA). The available treatments pose a burden of major adverse effects and new treatment strategies are therefore the need of time. In this study, *Pediococcus acidilactici* NMCC-B (Probiotic) was evaluated for its safety and efficacy in complete Freund’s adjuvant (CFA)-induced mice model of RA. Mice were treated with either *Escherichia coli* (1 × 10^9^ CFU/ml) or *P. acidilactici* NMCC-B (1 × 10^9^ CFU/ml, 2 × 10^9^ CFU/ml) to assess acute, sub-acute, and chronic toxicities. In RA model, mice were either pre-treated with daily dose of *P. acidilactici* NMCC-B or treated concurrently (day 1–day 27) or post-treated (day 28–day 42). *P. acidilactici* NMCC-B inhibited gut permeability, lessened joint inflammation, and ameliorated RA progression. No signs of toxicity, pathogenicity or bacterial translocation were observed in animals treated with probiotic. *P. acidilactici* NMCC-B also restored total body weight, attenuated inflammation, improved antioxidants, alleviated soft tissue swelling, bone damage, and the expression of IL-1β, NF-κB and TNF-α in paw tissue. Based on current findings, it is perceivable that *P. acidilactici* NMCC-B could be a promising candidate for the management of RA.

## Introduction

Rheumatoid arthritis (RA) is an autoimmune disability associated with inflammation of synovium, hyperplasia, production of autoantibodies like rheumatoid factor (RF) and anti-citrullinated protein antibody (ACPA), cartilage and bone deformity, and systemic manifestations such as cardiovascular, pulmonary, psychological and skeletal disorders. It also involves extra-articular complications, like rheumatoid nodules, vasculitis, and other systemic comorbid conditions [1].

Many therapeutic options are available to treat joint and cartilage damage with the outcome target of remission of RA. Over the past 30 years, various non-pharmacological and pharmacological resources have been implicated in alleviating the disability related to RA. Currently available pharmacological treatment approaches include analgesics, non-steroidal anti-inflammatory drugs (NSAIDs), corticosteroids, conventional synthetic and biological disease modifying anti-rheumatic drugs (DMARDs) [2]. The available pharmacotherapy is associated with severe adverse effects burden and impose risks to the quality of life (QOL) of a patient. Several NSAIDs have been used for the treatment of RA, which include naproxen, indomethacin, meloxicam etc., but all of them are associated with increased risk of gastrointestinal adverse effects [3]. Corticosteroids especially dexamethasone, prednisolone and hydrocortisone are responsible for immunosuppression in patients with RA [4]. Similarly, DMARDs like methotrexate causes patient non-compliance and approximately 20–30% of RA patients are unable to continue drug for more than 12 months due to adverse drug reactions (ADRs). Other DMARDs have also shown several ADRs which include GIT toxicity, hepatotoxicity, nephrotoxicity, and various blood-related toxicities [5]. Therefore, newer pharmacological treatments with less ADRs are required for the treatment of RA and to improve QOL of patients.

It has been reported that the dysbalanced gut microbiome leads to the initiation and perpetuation of RA [6]. Therefore, focusing on alternative treatments such as probiotics could be a better strategy as they are much safer than the current treatments. According to world health organization (WHO), probiotics are live, non-pathogenic microorganisms that confer health and well-being to the host when administered properly in a suitable amount. Probiotics are nutritional supplements that are generally considered safe with some therapeutic advantages [7]. The probiotic bacteria have the capability to maintain microbial equilibrium by replacing harmful microbes with beneficial bacteria. Owing to immunomodulatory potentials and safety profiles, probiotics have become an important source of alternative and complementary medicines in the current medical therapies. The two most commonly employed lactic acid-producing genera, *Lactobacillus* and *Bifidobacterium* species are effective therapeutic agents against inflammation in many immunological disorders [8].

In the current study, *Pediococcus acidilactici* NMCC-B is used as pharmacological treatment against CFA-induced mice model of RA. *P. acidilactici* NMCC-B is a non-spore-forming, gram-positive and lactic acid producing bacterium (LAB). These are rod-shaped, non-motile, catalase-negative, and facultative bacteria. Studies have shown that *P. acidilactici* NMCC-B produces bacteriocin, which act as antimicrobial peptides (AMPs), called “Pediocin”. These AMPs inhibit the growth of pathogenic bacteria and have a beneficial role on the human host by regulating the composition of gut microbiota, improving gut barrier function and host immunological response [9]. *P. acidilactici* NMCC-B also releases bacterial exopolymeric substances (EPS) which have diverse pharmacological properties such as anti-cancer, anti-hyperlipidemic, anti-oxidant, immunomodulatory, and anti-ulcerative. These substances show anti-inflammatory and anticancer properties by regulating apoptotic and nuclear factor-kappa B (NF-κB) pathways [10]. Moreover, EPS protects the bacteria from phagocytosis and exsiccation, thereby, assisting in forming a biofilm. At the same time, these EPS are responsible for decreasing the pathogenic biofilm production by bacteria [11].

Studies have shown that administration of LAB helps attenuate the interleukin (IL)-6 levels in RA patients. Treatment with *Lactobacillus* probiotics for 28 days in animal model has shown remission in arthritic scores. *Lactobacillus* species have also demonstrated a reduction in pro-inflammatory cytokines like IL-1β, IL-6, IL-17, and tumor necrosis factor-alpha (TNF-α), and elevation of anti-inflammatory cytokines, such as IL-4 and IL-10 [5]. In the present research study, the safety, efficacy and mechanism of action of *P. acidilactici* NMCC-B were assessed in complete Freund’s adjuvant (CFA) induced RA model.

## Materials and methods

### Materials

Chemicals and reagents used in the current study include complete Freund’s adjuvant (Sigma-Aldrich, Germany), dexamethasone (Decadron, OBS Pakistan), [glutathione, ascorbic acid, trichloroacetic acid (TCA), thiobarbituric acid (TBA), 5–5′dithio-bis-2-nitro benzoic acid (DTNB), 1-chloro-2,4-dinitrobenzene (CDNB), Griess reagent, bovine serum albumin (BSA), potassium sodium tartarate, copper sulphate and potassium iodide (Sigma Aldrich, Germany)].

### Animals

Male albino mice (BALB/c), 6–7 weeks old (20–25 g) were obtained from the National Institute of Health (NIH), Islamabad, Pakistan, and were kept for period of acclimatization (5–7 days). They were housed in the light-dark cycle for 12/12 hours and placed in plastic cages at 25 ± 2°C and humidity (55%) in the animal house of Department of Pharmacy, Quaid-i-Azam University, Islamabad, Pakistan. Mice were fed with standard feed and water. The experimental study was conducted according to ARRIVE guidelines and the approval was taken from the Bio-Ethical Committee (BEC) of Quaid-i-Azam University Islamabad (Approval number BEC-FBS-QAU2021–341).

### Probiotic strain

The probiotic bacterium *P. acidilactici* NMCC-B was collected from National Institute for Genomics and Advanced Biotechnology (NIGAB), National Agricultural Research Center (NARC), Islamabad Capital Territory, Pakistan. The dose of *P. acidilactici* NMCC-B was prepared under aseptic conditions in a laminar flow hood.

### Study design

*Toxicity study*: Safety assesment comprised of 21 days in which acute (day 7), sub-acute (day 14), and chronic (day 21) toxicity testing were conducted. Animals were daily observed for any toxicity signs or other related behavioral changes, including tremors, restlessness, weight loss, diarrhea, sluggishness, and paralysis during the study period. Mice were randomly divided into four groups after acclimatization, with 15 animals in each group (n = 15). Normal control group received no treatment, negative control group received *E. coli* (1 × 10^9^ CFU/ml/day), single dose probiotic group received single dose of *P. acidilactici* NMCC-B (1 × 10^9^ CFU/ml/day), and double dose probiotic group received double dose (2 × 10^9^ CFU/ml/day) orally Q.D. for 7, 14 and 21 days. The details of each experimental group are explained below:

For acute toxicity study: Total number of animals used = 60
**Group 1: Normal Control** (No treatment)
**Group 2: Negative Control** (*E. coli,* 1 × 10^9^ CFU/ml/day)
**Group 3: Single Dose** (*P. acidilactici* NMCC-B, 1 × 10^9^ CFU/ml/day)
**Group 4: Double Dose** (*P. acidilactici* NMCC-B, 2 × 10^9^ CFU/ml/day)

Similarly, for sub-acute and chronic studies, animals were divided into similar four groups with sixty animals for each study.

Upon completion of study periods, all the animals (180) were euthanized by intra-peritoneal (i.p.) injection of pentobarbital sodium (200 mg/kg) on day 7, 14 and 21. Care was taken to minimize the sufferings of animals. A well trained laboratory technician euthanized the animals according to standard protocols. Parameters such as respiratory depression and fainting of heart beat were used for the decision of euthanization during study period [12]. None of the animals died during the study period. Blood, serum, and organs were collected for subsequent analyses.

*CFA induced RA model*: The CFA-induced RA model consisted of 42 days. After the period of acclimatization, RA was induced with intra plantar injection of CFA (Ag; 20 μl) at day 0. On day 21, animals were first re-exposed to antigen (Ag; 0.1 ml/mice), and later exposed again to Ag on day 28, to check the immune system mobilization. The experimental animals (n = 5 in each group) were divided into six major groups: Normal control, negative control, positive control, pre-treatment, concurrent, and post-treatment group. The normal control group received simple water and feed ad libitum. The negative control group received 20 μl of CFA through intraplantar route (i.pl.). The positive control group received daily dose of dexamethasone (5mg/kg) via i.p. route. Pre-treatment group started receiving the daily dose of *P. acidilactici* NMCC-B (1 × 10^9^ CFU/ml/day) orally a week before induction. The concurrent group received treatment of *P. acidilactici* NMCC-B (1 × 10^9^ CFU/ml/day) simultaneously with the induction, i.e., Day 1 - Day 27. Post-treatment group received daily dose of *P. acidilactici* NMCC-B (1 × 10^9^ CFU/ml/day) from day 28 to day 42, after second exposure to Ag (CFA). Animal grouping and experimental study design is given below:

**Group 1: Normal Control** (Simple water and feed *ad libitum*)
**Group 2: Negative Control** (20 μl CFA, i.pl.)
**Group 3: Positive Control** (Dexamethasone, 5mg/kg, i.p.)
**Group 4: Pre-treatment** (*P. acidilactici* NMCC-B, 1 × 10^9^ CFU/ml/day, orally, from -7 day)
**Group 5: Concurrent** (*P. acidilactici* NMCC-B, 1 × 10^9^ CFU/ml/day, orally, simultaneously)
**Group 6: Post-treatment** (*P. acidilactici* NMCC-B, 1 × 10^9^ CFU/ml/day, orally, from day 28)

Upon completion of study period, all animals (30) were euthanized by using pentobarbital sodium (200 mg/kg) on day 14, 21, 28, 35 and 42. Parameters such as respiratory depression and fainting of heart beat were used for the decision of euthanization during study period [12]. None of the animals died during the study period. A well trained laboratory technician euthanized the animals according to standard protocols. Blood, serum, and organs were collected for subsequent analyses.

### Blood and serum collection

Blood was directly drawn from the heart by cardiac puncturing and collected immediately in EDTA tubes. A small portion of blood was collected in 1.5 ml flat bottom eppendorf tubes and centrifuged at 8000 RPM for 8 minutes to separate the yellowish-colored serum. Serum was cryopreserved immediately at -25 ºC for further analysis. EDTA tubes containing blood were processed for complete blood count (CBC) testing. The cryopreserved serum was used to perform serum biochemistry analysis.

### Isolation of organs

Animal were dissected following blood collection and organs such as liver, kidney, spleen, colon, feces, and paw were isolated, weighed on a digital weighing balance and stored in separate eppendorf tubes at -25 ºC. From these cryopreserved organs, homogenates were prepared by taking equal ratios of the organs and adding 1 ml of phosphate-buffered saline (PBS; pH = 7.4) in 2 ml of round bottom Eppendorf tubes. Homogenization was performed at 5800 RPM for 3–6 minutes, supernatant was separated and stored at -25 ºC for antioxidant assays. Isolated organs were also kept in 10% formalin for histopathological analysis.

### Body weight variations

The total body weight of the animals was recorded daily during the whole study. Changes in body weight were noted to analyze weight gain or weight loss [13]. These variations assisted in evaluating the nutritional role of *P. acidilactici* NMCC-B and the effect of RA on body weight.

### Relative organ weights

Organ weights of the mice were noted with the help of digital weighing balance on day 7, 14, and 21 of the safety study and on day 14, 21, 28, 35, and 42 of RA study. The relative weights of liver, kidney, and spleen were determined by using the following formula:

Relative organ weights = Weight of the organ/Total body weight × 100 [14]

### Paw thickness

To evaluate the arthritic index, paw edema or paw thickness parameter was measured using a standard digital thickness gauge meter. Arthritic score (0–4) was given to each experimental group based on the measurement of paw thickness [15]. Arthritic scoring was done as per mentioned below.

0 = No swelling or inflammation
1 = Slight swelling
2 = Erythema with mild edema
3 = Pronounced edematous swelling
4 = Joint rigidity

### Thermal hyperalgesia

Hot-plate test was used to measure thermal hyperalgesia. Experimental animals were kept on a hot plate with a temperature of 50–55 ºC for 25 seconds. Time latency to ‘paw licking’ and time latency to ‘jumping’ was noted [15].

### Muscle strength and coordination

For the determination of muscle strength and coordination inverted mesh test was performed. During this analysis, mice were placed in the middle of a mesh, which was held 40–50 cm above ground level. Mesh was then inverted for 60 seconds. Time latency to fall in seconds was recorded [16].

### Mechanical allodynia

Von frey test was conducted for the assessment of mechanical nociception in mice. First, acclimatized the mice to the wire grid of von frey apparatus for about 30–60 minutes. Then von frey filaments were placed onto the mid-plantar surface of the right hind paw with increasing force until the filament bent (~2–3 seconds). The paw withdrawal threshold was estimated by observing the response indicated by flicking and licking of the stimulated paw [16].

Serum biochemistry analysis – See Supplementary Methods
Estimation of antioxidants – See Supplementary Methods

### Radiological assessment

A radiological assessment of the mice paw was done at day 42 of the arthritis model to evaluate the joint deterioration and bone erosion.

### Histopathological analysis

Histopathological analysis was performed using hematoxylin and eosin staining. This examination was done with liver, kidney, spleen, and colon in the safety study, while in case of RA model, the investigated organs were liver, kidney, spleen, colon, and paw at different sampling points.

### Immunohistochemistry

The immunohistochemistry assay was performed to investigate the effects of inflammatory markers in CFA-induced arthritis model. Briefly, slides were first dipped in xylene for 5 minutes, followed by alcohol (100%, 90%, 80%, and 70%). They were later dipped in distilled water followed by PBS for 5 minutes. A drop of Proteinase K was added, and slides were dipped in PBS for 5 minutes. H_2_O_2_ solution (35%) was added for 10 minutes, followed by 5 minutes dipping in PBS. Normal goat serum (10 μl) was added, and slides were refrigerated for 90 minutes. Then, 10 μl of the primary antibody was added and slides were refrigerated overnight. On the second day of processing, slides were washed by PBS for 10 minutes and later 10 μl of secondary antibody (specific for NF-κB, IL-1β, and TNF-α) was applied for 2 hours and slides were refrigerated for 75 minutes. Subsequently, washing with PBS was carried out for 5 minutes and ABC (avidin-biotin complex) was applied for 75 minutes. Slides were again immersed in PBS, 100% alcohol, and xylene for 5 minutes each. Lastly, mounting media was added with a dropper and a coverslip was placed. Slides were stained by dipping in DAP dye to give brown color and air-dried on paper. Slides were allowed to dry overnight and observed under a microscope at 10X magnification [17].

### Statistical analysis

All data are expressed as mean ± SE. However, for analysis of discrete data, it was transformed to continuous data by taking LOG_10_ of the data for the calculation of mean standard error. All means were compared by two-way analysis of variance (ANOVA) and a LSD test was applied to compare (α = 0.05) the results using IBM SPSS 25 (SPSS for Windows, v25.0. SPSS Inc., Chicago, IL).

## Results

### Bacterial identification

#### *P. acidilactici* NMCC-B was identified by the following identification tests.

**Fig 1:**
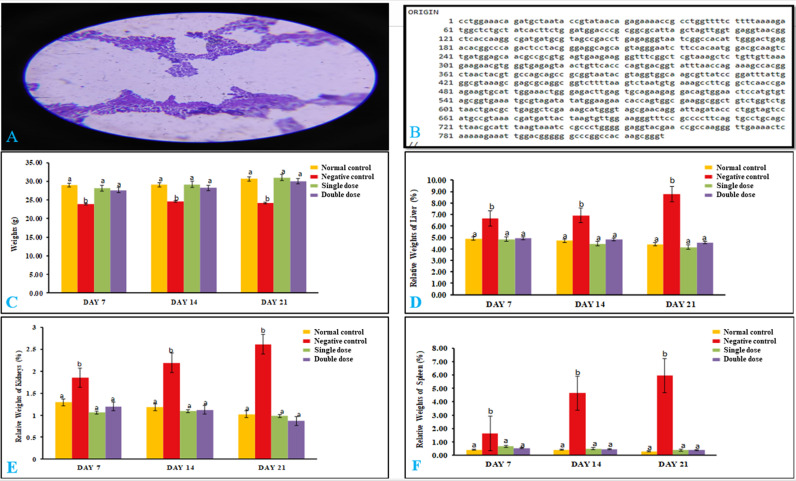
Bacterial identification and safety study of *P. acidilactici* NMCC-B. (A) Gram-staining of *P. acidilactici* NMCC-B. (B) *P. acidilactici* NMCC-B 16S Ribosomal RNA gene partial sequencing. (C) Total body weight of the safety study. (D) Relative weight of liver. (E) Relative weight of kidney. (F) Relative weight of spleen. *P. acidilactici* NMCC-B did not affect the body weight, organ weight or macroscopic appearance of the organs. Two way ANOVA followed by LSD for multiple comparisons (n = 15). The groups on the same day having different alphabetical superscripts are significantly different (p < 0.05). Negative control: *E. coli*, Single dose: *P. acidilactici* NMCC-B; Double dose: *P. acidilactici* NMCC-B.

*Gram staining*: The gram staining results showed purple-colored, cocci-shaped, gram-positive *P. acidilactici* NMCC-B (Fig 1A).

*Catalase test*: This test was used to identify bacteria that produce the enzyme catalase. The bubbles did not produce, which indicated catalase negative results for P. acidilactici NMCC-B.

*16S Ribosomal RNA sequencing*: The partial bacterial sequencing of *P. acidilactici* NMCC-B 16S ribosomal RNA gene was confirmed by gene sequencing (Fig 1B).

### Safety study

#### *P. acidilactici* NMCC-B did not affect the total body weight, relative organ weights and macroscopic appearance of internal organs.

There was no significant change in the body weights of the normal control group. The body weights of the negative control group decreased significantly as compared to the normal control group, while animals receiving single and double dose of *P. acidilactici* NMCC-B exhibited higher body weights as compared to negative control group (Fig 1C). Relative weights of liver, kidney, and spleen of *E. coli* group showed a drastic increase which represented the signs of hepatomegaly, nephromegaly, and splenomegaly. Conversely, the groups receiving a single and double dose of *P. acidilactici* NMCC-B did not show any significant changes in the mice organ weights compared to the normal control (Fig 1D–F). Internal organs of mice were assessed macroscopically at the end of the safety study (Day 21). The liver, kidneys, spleen, heart, lungs, and small and large intestine showed no external changes in physical appearances. The external scrutiny did not manifest any gross abnormalities (Fig S1).

#### *P. acidilactici* NMCC-B did not show any signs of toxicity on hematological and biochemical parameters.

In normal control group, no significant difference was observed in TLC, RBC, hemoglobin (Hb), platelet (PLT), lymphocyte, and granulocyte count. However, in the negative control group, there were elevated levels of WBCs, PLT along with reduced levels of RBCs, and Hb. Treatment with single and double dose of *P. acidilactici* NMCC-B did not show any significant alterations in hematological parameters, indicating no signs of toxicity or inflammation (Table 1).

**Table 1:** Effects of *E. coli* and *P. acidilactici* NMCC-B treatments on hematological parameters.

Parameters	Days	Normal control	Negative control (*E. coli*)	Single Dose (*P. acidilactici* NMCC-B)	Double Dose (*P. acidilactici* NMCC-B)
**TLC** **(10**^**3**^**/mm**^**3**^)	7	8.12 ± 0.24^a^	20.00 ± 0.87 ^b^	7.94 ± 0.21 ^a^	9.92 ± 0.24 ^a^
	14	7.00 ± 0.87 ^a^	18.56 ± 0.59 ^b^	7.52 ± 0.78 ^a^	5.90 ± 0.16 ^a^
	21	8.00 ± 1.16 ^a^	21.64 ± 0.36 ^b^	7.34 ± 0.27 ^a^	5.40 ± 0.29 ^a^
**RBC (10**^**6**^**/mm**^**3**^)	7	7.45 ± 0.55 ^a^	7.46 ± 0.54 ^b^	7.64 ± 0.33 ^a^	7.75 ± 0.15 ^a^
	14	8.71 ± 0.78 ^a^	7.52 ± 0.32 ^b^	7.83 ± 0.44 ^a^	7.93 ± 0.40 ^a^
	21	8.25 ± 1.19 ^a^	7.20 ± 0.80 ^b^	7.98 ± 0.65 ^a^	8.76 ± 0.87 ^a^
**Hb (g/dl)**	7	12.3 ± 0.57 ^a^	12.5 ± 0.55 ^b^	15.4 ± 0.32 ^a^	12.4 ± 0.57 ^a^
	14	15.0 ± 0.75 ^a^	12.1 ± 1.62 ^b^	14.2 ± 0.09 ^a^	13.5 ± 1.04 ^a^
	21	14.2 ± 1.76 ^a^	10.8 ± 1.19 ^b^	13.3 ± 0.59 ^a^	12.4 ± 0.92 ^a^
**PLT (10**^**3**^**/mm**^**3**^)	7	573.0 ± 25.7 ^a^	745.0 ± 28.8 ^b^	1011 ± 7.02 ^a^	594.4 ± 35.0 ^a^
	14	670.6 ± 90.1 ^a^	894.2 ± 52.8 ^b^	628.4 ± 35.9 ^a^	647.6 ± 33.0 ^a^
	21	917.2 ± 318 ^a^	1254 ± 29.2 ^b^	566.2 ± 15.4 ^a^	545.6 ± 36.5 ^a^
**Lymphocyte** **(10**^**3**^**/mm**^**3**^)	7	5.49 ± 0.24 ^a^	17.28 ± 0.61 ^b^	5.40 ± 0.80 ^a^	5.09 ± 0.91 ^a^
	14	5.22 ± 0.56 ^a^	15.63 ± 0.61 ^b^	5.81 ± 0.60 ^a^	4.36 ± 0.12 ^a^
	21	8.07 ± 0.95 ^a^	12.64 ± 0.54 ^b^	5.55 ± 0.13 ^a^	4.29 ± 0.29 ^a^
**Granulocyte** **(10**^**3**^**/mm**^**3**^)	7	2.85 ± 0.16 ^a^	5.13 ± 0.29 ^b^	7.86 ± 0.17 ^a^	9.74 ± 0.23 ^a^
	14	1.22 ± 0.13 ^a^	6.82 ± 1.28 ^b^	0.79 ± 0.08 ^a^	0.68 ± 0.14 ^a^
	21	1.49 ± 0.31 ^a^	5.38 ± 0.36 ^b^	1.03 ± 0.02 ^a^	0.84 ± 0.05 ^a^

*Note*: Each value represents mean ± S.E. (n = 5), Two way ANOVA followed by LSD for multiple comparisons. The groups on the same day having different alphabetical superscripts are significantly different (p < 0.05).

The levels of total serum protein, albumin, and globulin were significantly low in negative control (*E.coli*) group, while the creatinine level was enhanced significantly at day 7, 14, and 21. Single and double dose of *P. acidilactici* NMCC-B had higher levels of total serum protein, albumin, and globulin, but this increase was not higher than the normal control. Likewise, creatinine level was less in the probiotic treated groups (Table 2).

**Table 2:** Effects of *E. coli* and *P. acidilactici* NMCC-B treatments on biochemical parameters.

Parameters	Days	Normal control	Negative control (*E. coli*)	Single Dose (*P. acidilactici* NMCC-B)	Double Dose (*P. acidilactici* NMCC-B)
**Albumin** **(g/dl)**	7	3.69 ± 0.33^a^	0.43 ± 0.05 ^b^	2.92 ± 0.07 ^a^	2.42 ± 0.08 ^a^
	14	3.45 ± 0.12 ^a^	0.51 ± 0.04 ^b^	3.23 ± 0.08 ^a^	3.26 ± 0.12 ^a^
	21	3.44 ± 0.15 ^a^	0.64 ± 0.02 ^b^	3.31 ± 0.09 ^a^	3.15 ± 0.13 ^a^
**Globulin (g/dl)**	7	1.77 ± 0.33 ^a^	0.75 ± 0.27	2.79 ± 0.05 ^a^	3.02 ± 0.10 ^a^
	14	3.15 ± 0.09 ^a^	1.94 ± 0.19 ^b^	2.26 ± 0.08 ^a^	2.04 ± 0.55 ^a^
	21	2.44 ± 0.33 ^a^	0.69 ± 0.14 ^b^	2.47 ± 0.11 ^a^	2.37 ± 0.41 ^a^
**Total protein (g/dl)**	7	5.47 ± 0.05 ^a^	3.18 ± 0.29 ^b^	5.71 ± 0.05 ^a^	5.44 ± 0.08 ^a^
	14	6.59 ± 0.08 ^a^	2.45 ± 0.23 ^b^	5.50 ± 0.06 ^a^	5.31 ± 0.62 ^a^
	21	5.88 ± 0.28 ^a^	1.33 ± 0.15 ^b^	5.78 ± 0.06 ^a^	5.52 ± 0.38 ^a^
**Creatinine (mg/dl)**	7	0.28 ± 0.130 ^a^	2.62 ± 0.13 ^b^	1.18 ± 0.08 ^a^	0.91 ± 0.14 ^a^
	14	0.22 ± 0.13 ^a^	2.29 ± 0.06 ^b^	0.75 ± 0.10 ^a^	0.64 ± 0.05 ^a^
	21	0.50 ± 0.16 ^a^	3.29 ± 0.11 ^b^	0.62 ± 0.08 ^a^	0.43 ± 0.09 ^a^

***Note***: Each value represents mean ± S.E. (n = 5), Two way ANOVA followed by LSD for multiple comparisons. The groups on the same day having different alphabetical superscripts are significantly different (p < 0.05).

#### *P. acidilactici* NMCC-B maintained antioxidant concentrations to normal levels.

**Fig 2:**
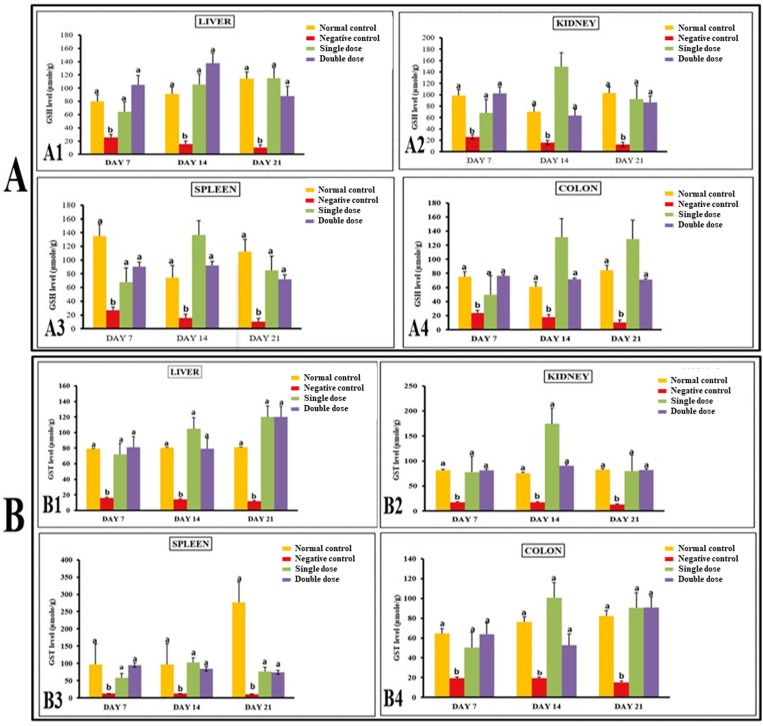
*P. acidilactici* NMCC-B did not attenuate the levels of GSH and GST. (A) GSH levels (μmole/g) in liver (A1), kidney (A2), spleen (A3), and colon (A4). (B) GST levels (μmole/g) in liver (B1), kidney (B2), spleen (B3), and colon (B4). Both doses of *P. acidilactici* NMCC-B significantly induced the levels of GSH and GST. Two-way ANOVA followed by LSD for multiple comparisons, (n = 5). The groups on the same day having different alphabetical superscripts are significantly different (p < 0.05). Negative control: *E. coli*, Single dose: *P. acidilactici* NMCC-B; Double dose: *P. acidilactici* NMCC-B.

**Fig 3:**
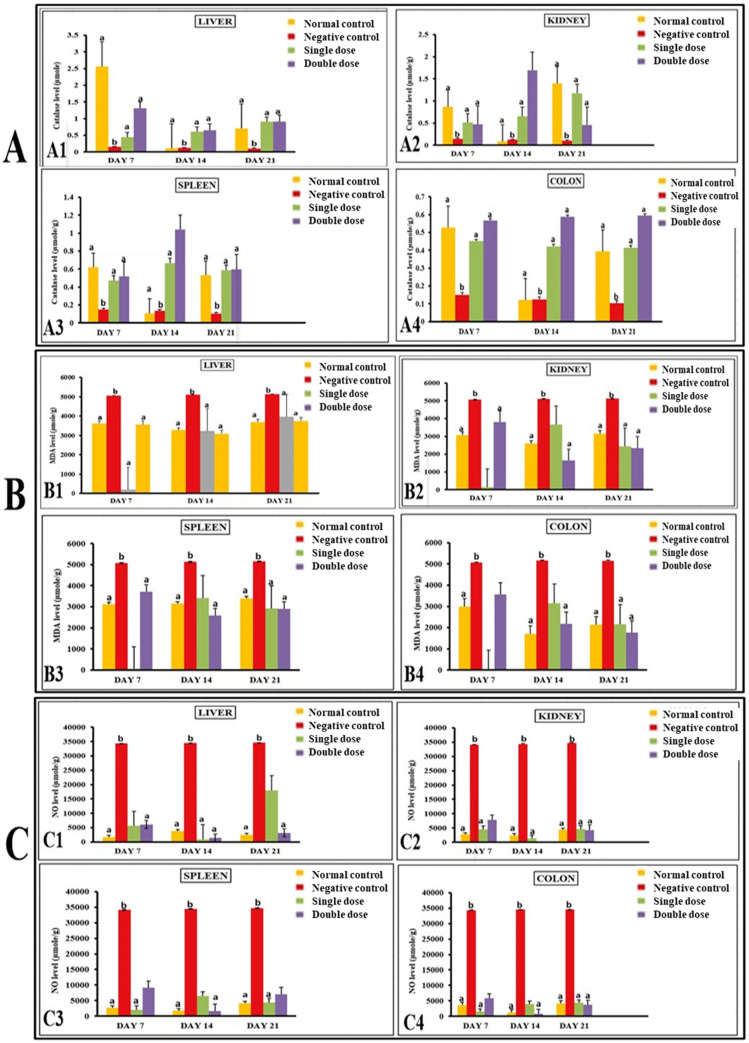
*P. acidilactici* NMCC-B stabilized the levels of catalase, MDA and NO. (A) Catalase levels (μmole/g) in liver (A1), kidney (A2), spleen (A3), and (A4) colon. (B) MDA levels (μmole/g) in liver (B1), kidney (B2), spleen (B3), and colon (B4). (C) NO levels (μmole/g) in liver (C1), kidney (C2), spleen (C3), and colon (C4). *P. acidilactici* NMCC-B significantly induced the levels of catalase along with a reduction in NO and MDA levels. Two-way ANOVA followed by LSD for multiple comparisons, (n = 5). The groups on the same day having different alphabetical superscripts are significantly different (p < 0.05). Negative control: *E. coli*, Single dose: *P. acidilactici* NMCC-B; Double dose: *P. acidilactici* NMCC-B.

Treatment with *E. coli* significantly reduced glutathione (GSH) levels in different organs of mice. In contrast, the levels of GSH in liver, kidneys, spleen, and colon were significantly higher in groups treated with single and double dose of P. acidilactici NMCC-B. Similarly, negative control (E. coli) group showed a significant reduction in gluthione-S-transferase (GST) and catalase levels as compared to normal control group. The levels of GST and catalase in various organs were significantly enhanced in groups receiving P. acidilactici NMCC-B (Figs 2 and 3).

Moreover, the E.coli group showed elevated levels of malondialdehyde (MDA) and nitric oxide (NO) in comparison to normal control group. MDA and NO concentrations in liver, kidneys, spleen, and colon were significantly low in groups receiving single and double dose of P. acidilactici NMCC-B (Fig 3).

### *P. acidilactici* NMCC-B demonstrated normal histology of various organs

**Fig 4:**
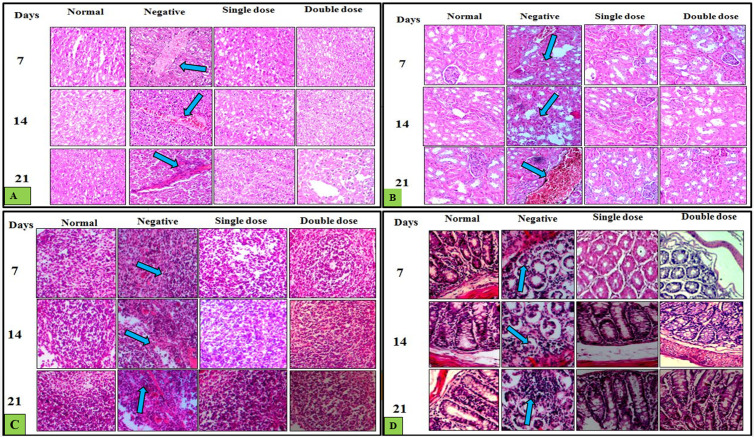
Histopathological analysis reiterated the safety profile of *P. acidilactici* NMCC-B. (A) Histopathology of liver: The arrows represent abnormal hepatocytes, RBCs congestion, and cellular infiltration. (B) Renal histopathology: The arrows represent deteriorated glomerulus, hypercellularity, and capillary aneurysm. (C) Histopathology of spleen: The arrows are representing white pulp atrophy and red pulp hyperplasia. (D) Histopathology of colon: The arrows represent goblet cell depletion, loss of crypts, and infiltration. Animals treated with *P. acidilactici* NMCC-B did not show any significant histological alterations in investigated organs.

Histopathology of liver in the normal control group showed no major changes. The cellular architecture was perfect, with normal central veins and hepatocytes. In negative control (*E. coli*) group, there was increased cellular infiltration with abnormal hepatocytes architecture at day 21. RBCs congestion was observed in the negative group. However, in groups receiving a single and double dose of *P. acidilactici* NMCC-B, no such abnormalities were observed (Fig 4A). Likewise, renal histopathology of the normal control group showed intact glomerulus, normal architecture of blood vessels and Bowman’s capsule. The negative control group showed deteriorated glomerulus and capillary aneurysm. Groups receiving single and double dose of *P. acidilactici* NMCC-B exhibited normal glomerulus with no cellular infiltration (Fig 4B).

Histopathological analysis of spleen in normal control group showed normal splenic architecture with clear red and white pulp distinction. *E. coli* group exhibited atrophy of the white pulp along with the hyperplasia of the red pulp. Moreover, *P. acidilactici* NMCC-B treated groups demonstrated mature erythrocytes in the red pulp and normal white pulp splenic sections (Fig 4C). Histopathology of colon in normal control group showed no inflammation or cellular infiltrates within the mucosa and sub-mucosa. However, the negative control group resulted in severe inflammation with epithelial hyperplasia, goblet cell depletion and ulceration. Groups with a single and double dose of *P. acidilactici* NMCC-B exhibited normal colonic architecture (Fig 4D).

### CFA-induced RA study

#### *P. acidilactici* NMCC-B alleviated CFA-induced paw edema, arthritic index and behavior impairment.

**Fig 5:**
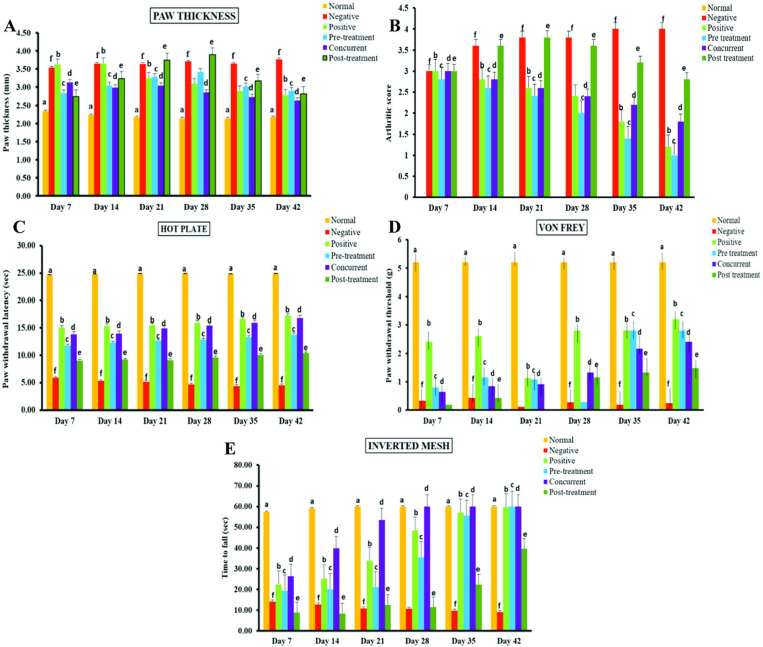
*P. acidilactici* NMCC-B improved paw edema, arthritic index and behavioral parameters. (A) Effect of *P. acidilactici* NMCC-B on paw thickness. (B) Effect of *P.* on the arthritic index. (C) Effect of *P. acidilactici* NMCC-B on paw withdrawal latency. (D) Effect of *P. acidilactici* NMCC-B on paw withdrawal threshold. (E) Effect of *P. acidilactici* NMCC-B on time latency to fall. Two way ANOVA followed by LSD for multiple comparisons (n = 5). *P. acidilactici* NMCC-B significantly reduced paw inflammation, arthritic index and improved paw withdrawal latency and threshold along with muscle strength and coordination. The groups on the same day having different alphabetical superscripts are significantly different (p < 0.05). Normal - No treatment, Negative - CFA treatment, Positive - Dexamethasone treatment, Pre-treatment/Concurrent/Post-treatment - *P. acidilactici* NMCC-B.

The administration of CFA into the mid-plantar surface of the mice paw produced significant macroscopic changes like swelling of the paw, severe inflammation, and erythema. However, treatment with *P. acidilactici* NMCC-B lessened swelling and abolished erythema. Treatment with dexamethasone also reduced paw edema/thickness to some extent (Fig 5A).

Arthritic index represents severity of the disease and is calculated on the basis of clinical arthritic score, as mentioned in method section. Treatment with *P. acidilactici* NMCC-B significantly improved arthritic index compared to the CFA administered group (Negative control). The pre-treatment group showed the least arthritic index among all probiotic groups which indicates that *P. acidilactici* NMCC-B could be highly effective in reducing the progression of RA (Fig 5B).

The hot-plate test evaluated the thermal pain reflexes by footpad contact with a heated surface. Treatment with *P. acidilactici* NMCC-B remarkably increased the latency time of paw withdrawal (Fig 5C)**.** Furthermore, administration of CFA significantly enhanced the sensitivity towards allodynic stimuli or pain threshold in von Frey test. Treatment with *P. acidilactici* NMCC-B markedly improved pain nociception in mice. Animals receiving *P. acidilactici* NMCC-B post-treatment showed enhanced reduction in sensitivity towards allodynic stimuli, and elevated paw withdrawal threshold as compared to concurrent and pre-treatment (Fig 5D). An inverted mesh test was employed to assess muscle strength coordination post-CFA treatment. *P. acidilactici* NMCC-B treatment did not exhibit any major alterations in strength and coordination. However, the probiotic group significantly improved these conditions compared to the CFA-treated group. On day 42, groups receiving *P. acidilactici* NMCC-B either prophylactically or concurrently displayed maximum latency time with the highest muscle strength and coordination (Fig 5E).

#### *P. acidilactici* NMCC-B improved detrimental effects of CFA on body weight and relative organ weights.

The relative organ weights of normal control group did not show any significant alterations. CFA-treated animals showed a significant increase in the relative weight of liver, kidney, and spleen which showed the signs of hepatomegaly, nephromegaly, and splenomegaly. However, animals receiving dexamethasone and *P. acidilactici* NMCC-B helped in attenuating inflammation of organs. Moreover, there was no significant difference in the total body weight of the normal control group. The total body weight of the CFA-administered group was significantly decreased. On the other hand, animals receiving *P. acidilactici* NMCC-B as prophylactic treatment and concurrent treatment showed a significant increase in the total body weight at day 42. Similarly, animals in the post-treatment and positive control (dexamethasone) groups also exhibited a slight increase in total body weight as compared to negative control (CFA treated) group (Fig S2).

#### *P. acidilactici* NMCC-B improved the levels of antioxidants in RA model.

Antioxidant assay demonstrated that the negative (arthritic) control group had significantly reduced GSH levels compared to the normal control group. On day 42, animals receiving *P. acidilactici* NMCC-B and dexamethasone exhibited a significant increase in GSH levels in various tissues. The pre-treatment group showed maximum antioxidant effect as compared to concurrent and post-treatment. Similarly, the CFA-treated group had significantly reduced GST as compared to the normal control group. The positive (dexamethasone) control group showed slight increase in GST content. Animals receiving *P. acidilactici* NMCC-B showed a significantly increased quantity of GST (Fig S3).

Treatment with *P. acidilactici* NMCC-B also increased the enzyme activity of catalase. Especially the group that received *P. acidilactici* NMCC-B as a prophylactic treatment showed the maximum activity of antioxidant-catalase. In comparison, the level of catalase enzyme was significantly decreased in the arthritic control group. Moreover, the CFA-treated group had significantly increased MDA levels compared to the normal control group. Animals receiving dexamethasone also showed a slight MDA elevation compared to the normal control group. The groups that received *P. acidilactici* NMCC-B had slight improvement in ameliorating the levels of MDA (Fig S4). CFA induced oxidative stress significantly increased NO levels in the negative control group. In comparison, the probiotic and dexamethasone treated groups reduced NO levels, thereby, helped in alleviating oxidative stress (Fig S5).

#### Histopathological analysis revealed protective and curative effects of *P. acidilactici* NMCC-B.

**Fig 6:**
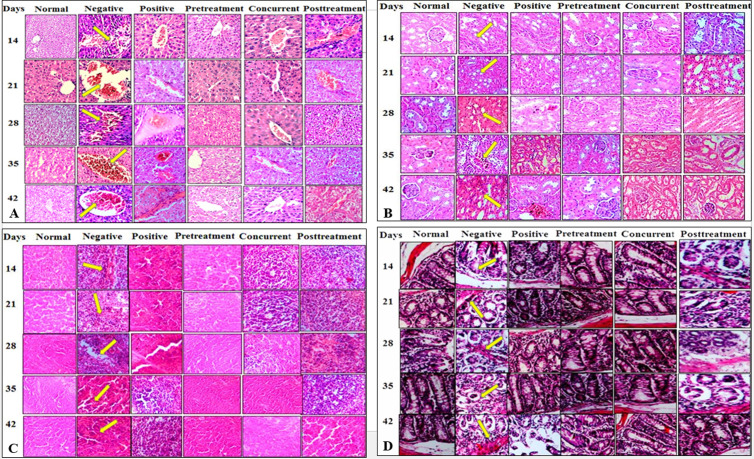
P. acidilactici NMCC-B protected against CFA induced histological abnormalities. (A) Histopathology of liver: The arrows represent abnormal hepatocytes, RBCs congestion, and cellular infiltration (B) Histopathology of kidney: The arrows represent deteriorated glomerulus, hypercellularity, and capillary aneurysm (C) Histopathology of spleen: The arrows are representing white pulp atrophy, red pulp hyperplasia, and infiltration in lymphoid follicles (D) Histopathology of colon: The arrows represent goblet cell depletion, loss of crypts, and cellular infiltration. Prophylactic treatment with *P. acidilactici* NMCC-B effectively ameliorated the inflammation and associated histological abnormalities as compared to concurrent or post-treatment. Normal - No treatment, Negative - CFA treatment, Positive - Dexamethasone treatment, Pre-treatment/Concurrent/Post-treatment - *P. acidilactici* NMCC-B.

The histopathological evaluation of liver in the normal control group showed normal central vein and hepatocytes. The CFA-treated group showed a significant cellular infiltration with abnormal hepatocyte architecture. RBCs congestion was observed in the CFA-arthritic control group. The positive control (dexamethasone) group also showed some deleterious effects such as abnormal hepatic architecture. RBCs congestion was seen in the portal vein. However, treatment with *P. acidilactici* NMCC-B showed a significant reduction in hepatic infiltration. Prophylactic treatment of *P. acidilactici* NMCC-B proved effective in controlling majority of the abnormalities (Fig 6A).

Renal histopathological evaluation of the normal control group exhibited normal architecture of blood vessels, intact glomerulus, and Bowman’s capsule. The CFA-treated animals showed significant destruction of the glomerulus. CFA caused severe cellular infiltration, edema exudate, and necrotic foci. Hypercellularity and capillary aneurysm were observed in arthritic control group. The dexamethasone-treated animals showed distorted glomeruli and mild infiltration of inflammatory cells. In contrast, treatment with *P. acidilactici* NMCC-B helped in alleviating infiltration. Prophylactic treatment of *P. acidilactici* NMCC-B was crucial in maintaining the normal renal cortex and glomerular tufts by attenuating the inflammation caused by CFA (Fig 6B).

The histopathological analysis of spleen in the normal control group showed normal splenic architecture with typical lymphoid follicles and sinuses. The distinction between red and white pulp was cleared. The CFA-treated group depicted a significant deterioration of red and white pulp. Red pulp hyperplasia and white pulp atrophy were observed. On the other hand, animals treated with dexamethasone showed an ill-defined spleen section with infiltration in lymphoid follicles representing the signs of toxicity. In comparison, groups receiving *P. acidilactici* NMCC-B exhibited a significant decrease in inflammatory exudates. The splenic architecture of pre-treatment animals resembled normal control architecture. Post-treatment group was least effective in reducing the inflammation and maintaining the normal splenic structure (Fig 6C).

Histopathology of colon in the normal control group depicted no signs of inflammation within the mucosal layer of epithelium. The CFA-arthritic control group showed a significant increase in epithelial hyperplasia. Goblet cells were depleted, and crypts were shortened or lost in some areas of the colon due to CFA-induced inflammation. The intestinal damage was mild in the positive dexamethasone group, but disturbed crypt architecture with infiltration at the end of study was witnessed. Animals receiving *P. acidilactici* NMCC-B treatment showed normal colonic architecture. No crypt damage, no ulceration and no invasion of inflammatory cells were observed in any group receiving *P. acidilactici* NMCC-B. Prophylactic treatment with *P. acidilactici* NMCC-B proved to be more effective in ameliorating the inflammation as compared to concurrent or post-treatment (Fig 6D).

#### *P. acidilactici* NMCC-B protected against CFA induced RA.

**Fig 7:**
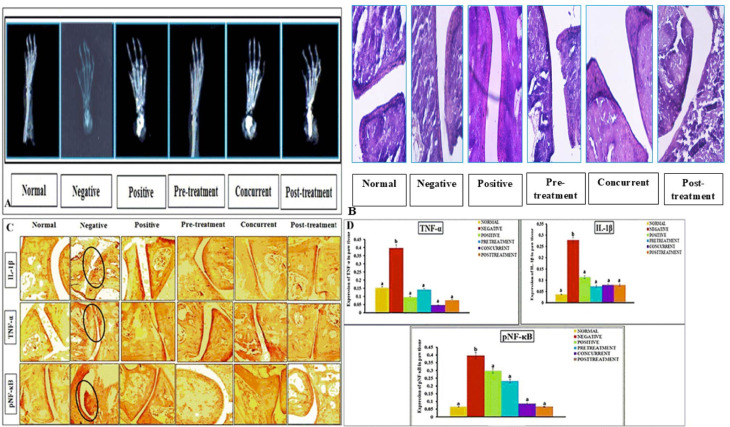
P. acidilactici NMCC-B protected CFA induced arthritic damage. (A) Radiographic examination of ankle joint of the mice paw at day 42 (B) Histopathology of mice paw, where arrows are representing synovial hyperalgesia, infiltration of pro-inflammatory cells into the synovial lining, narrowing of the joint space, pannus formation, bone erosion and cartilage deterioration (C) Effect of *P. acidilactici* NMCC-B on IL-1β, TNF-α, and NF-κB expression on day 42 (D) Quantitative analysis of IL-1β, TNF-α, and NF-κB. *P. acidilactici* NMCC-B efficiently reduced CFA induced arthritic damage and pro-inflammatory mediators. Two-way ANOVA followed by LSD for multiple comparisons. The groups on the same day having different alphabetical superscripts are significantly different (p < 0.05). Normal - No treatment, Negative - CFA treatment, Positive - Dexamethasone treatment, Pre-treatment/Concurrent/Post-treatment - *P. acidilactici* NMCC-B.

Histopathological evaluation of the ankle joint in the normal control group did not show any narrowing of joint space. Articular cartilage and synovium were normal with no inflammatory exudates. On day 35 and 42, the histopathology of CFA-treated group exhibited a significant increase in synovial hyperalgesia, marked elevation of infiltration of pro-inflammatory cells into the synovial lining, narrowing of the joint space, pannus formation, bone erosion and cartilage deterioration. However, dexamethasone showed mild inflammatory cell infiltration and cartilage damage with some extent of pannus formation. On the other hand, treatment with *P. acidilactici* NMCC-B exhibited a significant reduction in inflammatory cells, space narrowing, cartilage damage and bone erosion with no pannus development. Prophylactic administration of *P. acidilactici* NMCC-B resulted in noticeable improvements in the CFA-induced abnormalities. The cellular structure of paw tissue of the pre-treatment group resembled the normal architecture of the ankle joint compared with the post-treatment group (Fig 7A). Radiological examination of the normal control group also showed normal architecture of the ankle with no swelling around the joint. X-ray of the CFA-treated group exhibited marked morphological abnormalities. It showed a significant reduction in joint space that resulted in cartilage deterioration and bone erosion. The dexamethasone group showed mild soft tissue damage and bone erosion compared to the normal control. Treatment with *P. acidilactici* NMCC-B efficiently lessened soft tissue swelling, joint space narrowing, bone damage, and joint deformity (Fig 7B).

Immunohistochemical analysis displayed no expression of inflammatory markers in the normal control group. CFA administration resulted in the pronounced elevation of pro-inflammatory mediators. Expression of IL-1β, TNF-α, and NF-κB significantly increased in negative (CFA-treated) control group. Dexamethasone also showed mild expression of the inflammatory markers. However, on day 42, treatment with *P. acidilactici* NMCC-B markedly improved the inflammatory state and significantly reduced the expression of inflammatory mediators. Prophylactic treatment with *P. acidilactici* NMCC-B showed considerable improvements in ameliorating the expression of inflammatory markers in paw tissue (Fig 7C and D).

## Discussion

It is a prerequisite to ensure the safety of probiotic bacteria with the advent of new strains [18]. Since the market demand for probiotic bacteria are increasing tremendously, hence, their safety profile should be the top priority and must be ensured according to the qualified presumption of safety-EFSA [19,20]. LABs are generally considered safe for human consumption, but in some cases, *Lactobacilli* and *Bifidobacterium* can cause bacteremia, sepsis, meningitis, endocarditis and abscesses in immune-compromised patients. This may raise safety concerns and question their safety profile [21].

In current study, *in-vivo* safety of *P. acidilactici* NMCC-B was evaluated, utilizing single (1 × 10^9^ CFU/ml/day) and double (2 × 10^9^ CFU/ml/day) dose of probiotic, through acute, sub-acute, and chronic phases of oral toxicity testing. The general parameters of *in-vivo* safety evaluation included feed intake, signs of toxicity, gross examination, weight variations in total body weight and organ weights, hematology, serum biochemistry, antioxidants and oxidative stress markers, internal organs histology and bacterial translocations [22]. Both doses of *P. acidilactici* NMCC-B were safe; no mortality was seen, no significant weight loss was observed, not any pathological abnormality was shown in histology, and no bacterial translocation to internal organs and bacteremia was present. However, the negative (*E. coli*) group showed a drastic weight loss, enhanced TLC, platelets, lymphocytes, granulocytes count, serum creatinine, MDA and NO level and decreased serum albumin and globulin content, GSH, GST and catalase levels. *E.coli* group showed the signs of hepatomegaly, nephromegaly, and splenomegaly, and also histological abnormalities. These findings affirmed that *P. acidilactici* NMCC-B did not disturb the normal functioning of various organs and gut microbiome. The results of this study are in line with previous studies, which reports that dysbiosis of gut microbiome and inflammation cause oxidative stress, histological abnormalities and disturbance in hematological and biochemical parameters [22–25].

After assuring the *in-vivo* safety, the pharmacological role of *P. acidilactici* NMCC-B in the prevention or treatment of RA was evaluated. CFA was used to induce RA in mice [26], as CFA induced RA closely mimic many particular characteristics of human RA [27]. CFA is composed of an emulsion of heat-killed *Mycobacterium tuberculosis* in oil adjuvant and has ligands for many toll like receptors (TLRs) which upon activation results in the production of inflammatory modulators [28]. Intraplantar inoculation of CFA in hind paw of the mice generally stimulates the innate immune response and a severe inflammatory cascade [29]. Mycobacterium administration results in edema due to the migration of extracellular fluid and debris at the inflammation site and paw edema gradually gets worsen with the passage of time [30–32]. The present study also demonstrated macroscopic alterations like pronounced edematous swelling of the mice paw in arthritic control. Treatment with *P. acidilactici* NMCC-B significantly reduced the paw volume by inhibiting the inflammation and improved arthritic index by alleviating the disease severity and progression.

Studies have shown that CFA releases various inflammatory mediators that caused pain and enhanced sensitization of nociceptor receptors, leading to decreased pain thresholds [33–36]. In this study, thermal hyperalgesia, mechanical allodynia, and muscle strength assessment parameters were significantly decreased, following CFA administration and treatment with *P. acidilactici* NMCC-B significantly enhanced paw withdrawal latency in hot-plate, elevated allodynic withdrawal threshold via von frey assessment, and improved muscle strength and coordination. These beneficial effects of *P. acidilactici* NMCC-B might be attributed to its anti-inflammatory effects.

Another characteristic feature of rheumatism cachexia includes weight loss and appetite. CFA-arthritic mice exhibited a drastic body weight loss. This can be due to the production of a hormone, leptin, which results in low feed consumption and weight loss [37]. CFA also causes overexpression of pro-inflammatory cytokines that triggered proteolysis and muscle deterioration [38]. Moreover, CFA is known to increase the relative weight of internal organs due to edema caused by inflammatory mediators [26]. We also witnessed weight loss, hepatomegaly, nephromegaly, and splenomegaly in CFA treated mice. Treatment with *P. acidilactici* NMCC-B significantly reversed CFA induced abnormal alterations.

There has been a strong relationship between RA severity and oxidative stress. Production of reactive oxygen species (ROS) at the site of inflammation enhances with the progression of disease [39,40]. This imbalance in antioxidants and oxidants consequently leads to stress that demolishes the physiological functioning of biological macromolecules like lipid peroxidation, DNA degradation, and protein oxidation [11]. In this study, CFA-induced RA caused noticeable oxidative stress, which was manifested by the significant rise in MDA and NO levels and decrease in GSH, GST, and catalase levels. *P. acidilactici* NMCC-B treatment considerably alleviated the tissue damage by restoring the natural antioxidant defense system, which is in line with previous studies [41].

This study revealed that the administration of CFA induced inflammatory mediators and exacerbated immune response. The expression of pro-inflammatory cytokines such as TNF-α, NF-κB, and IL-1β, were augmented in the CFA-treated group, resulting in chronic inflammation. TNF-α is not only involved in the inhibition of regulatory T-cells functions but is also responsible for the phosphorylation and transcription of NF-κB. NF-κB, in turn, caused the manifestations of osteoclasts and bone resorption [33,42,43]. Current research findings elaborated that the treatment with *P. acidilactici* NMCC-B significantly reduced elevated expression of these pro-inflammatory cytokines, which indicates that its anti-arthritic effects could be due to suppression of pro-inflammatory mediators.

## Conclusion

It is conceivable from this study that *P. acidilactici* NMCC-B is safe and effective in reducing RA symptoms in mice. It decreased inflammation, pain, and oxidative stress while restoring antioxidant levels and body weight. The probiotic also lowered key inflammatory markers (TNF-α, NF-κB, IL-1β), suggesting its potential as a natural RA treatment.

## Supporting information

S1 Fig Macroscopic assessment of internal organs of mice treated with single and double dose of *P. acidilactici* at day 21. (a) Heart (b) Liver (c) Kidneys (d) Spleen (e) Stomach (f) Small intestine (g) Caecum (h) Colon. Treatment did not demonstrated any signs of abnormality in internal organs.
(PNG)

S2 Fig *P. acidilactici* improved body weight and reduced relative organ weight. (A) Relative weight of liver (B) Relative weight of kidney (C) Relative weight of spleen (D) Total body weight. Two way ANOVA followed by LSD for multiple comparisons (n = 5). The groups on the same day having different alphabetical superscripts are significantly different (p < 0.05). Normal – No treatment, Negative – CFA treatment, Positive – Dexamethasone treatment, Pre-treatment/Concurrent/Post-treatment – *P. acidilactici*.
(PNG)

S3 Fig *P. acidilactici* enhanced GSH and GST levels in RA model. (A) Effect of *P. acidilactici* on GSH level in liver (A1), kidney (A2), spleen (A3), colon (A4), and paw (A5). (B) Effect of *P. acidilactici* on GST level in liver (B1), kidney (B2), spleen (B3), colon (B4), and paw (B5). Two way ANOVA followed by LSD for multiple comparisons (n = 5). The groups on the same day having different alphabetical superscripts are significantly different (p < 0.05). Normal – No treatment, Negative – CFA treatment, Positive – Dexamethasone treatment, Pre-treatment/Concurrent/Post-treatment – *P. acidilactici*.
(TIF)

S4 Fig *P. acidilactici* improved catalase and MDA levels in RA model. (A) Effect of *P. acidilactici* on catalase level in liver (A1), kidney (A2), spleen (A3), colon (A4), and paw (A5). (B) Effect of *P. acidilactici* on MDA level in liver (B1), kidney (B2), spleen (B3), colon (B4), and paw (B5). Two way ANOVA followed by LSD for multiple comparisons (n = 5). The groups on the same day having different alphabetical superscripts are significantly different (p < 0.05). Normal – No treatment, Negative – CFA treatment, Positive – Dexamethasone treatment, Pre-treatment/Concurrent/Post-treatment – *P. acidilactici*.
(TIF)

S5 Fig *P. acidilactici* reduced NO levels in RA model. Effect of *P. acidilactici* on NO level in liver (A), kidney (B), spleen (C), colon (D), and paw (E). Two way ANOVA followed by LSD for multiple comparisons (n = 5). The groups on the same day having different alphabetical superscripts are significantly different (p < 0.05). Normal – No treatment, Negative – CFA treatment, Positive – Dexamethasone treatment, Pre-treatment/Concurrent/Post-treatment – *P. acidilactici*.
(TIF)
