# Supplementary figures and images for: Comprehensive safety assessment and therapeutic potential of Pediococcus acidilactici NMCC-B in attenuating arthritis progression
Source: PLoS One. 2025 May 22;20(5):e0324060. doi: 10.1371/journal.pone.0324060 (PMC12097640; doi:10.1371/journal.pone.0324060)

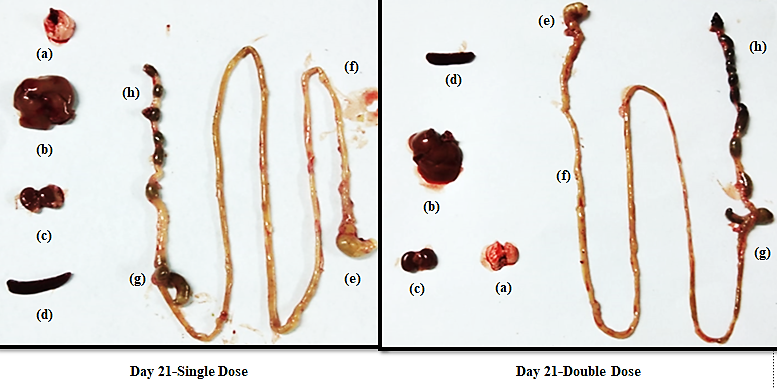

Supplement: S1 Fig — (a) Heart (b) Liver (c) Kidneys (d) Spleen (e) Stomach (f) Small intestine (g) Caecum (h) Colon. Treatment did not demonstrated any signs of abnormality in internal organs. (PNG) [file pone.0324060.s001.png]

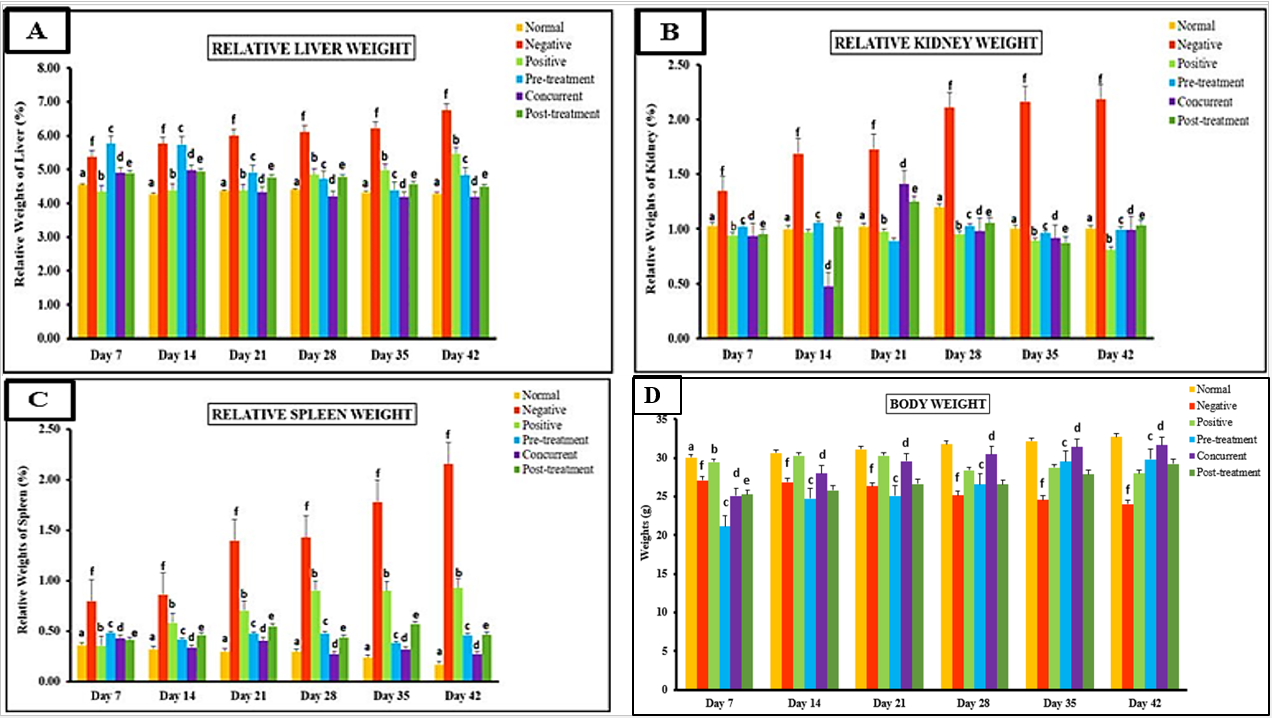

Supplement: S2 Fig — (A) Relative weight of liver (B) Relative weight of kidney (C) Relative weight of spleen (D) Total body weight. Two way ANOVA followed by LSD for multiple comparisons (n = 5). The groups on the same day having different alphabetical superscripts are significantly different (p < 0.05). Normal – No treatment, Negative – CFA treatment, Positive – Dexamethasone treatment, Pre-treatment/Concurrent/Post-treatment – P. acidilactici. (PNG) [file pone.0324060.s002.png]

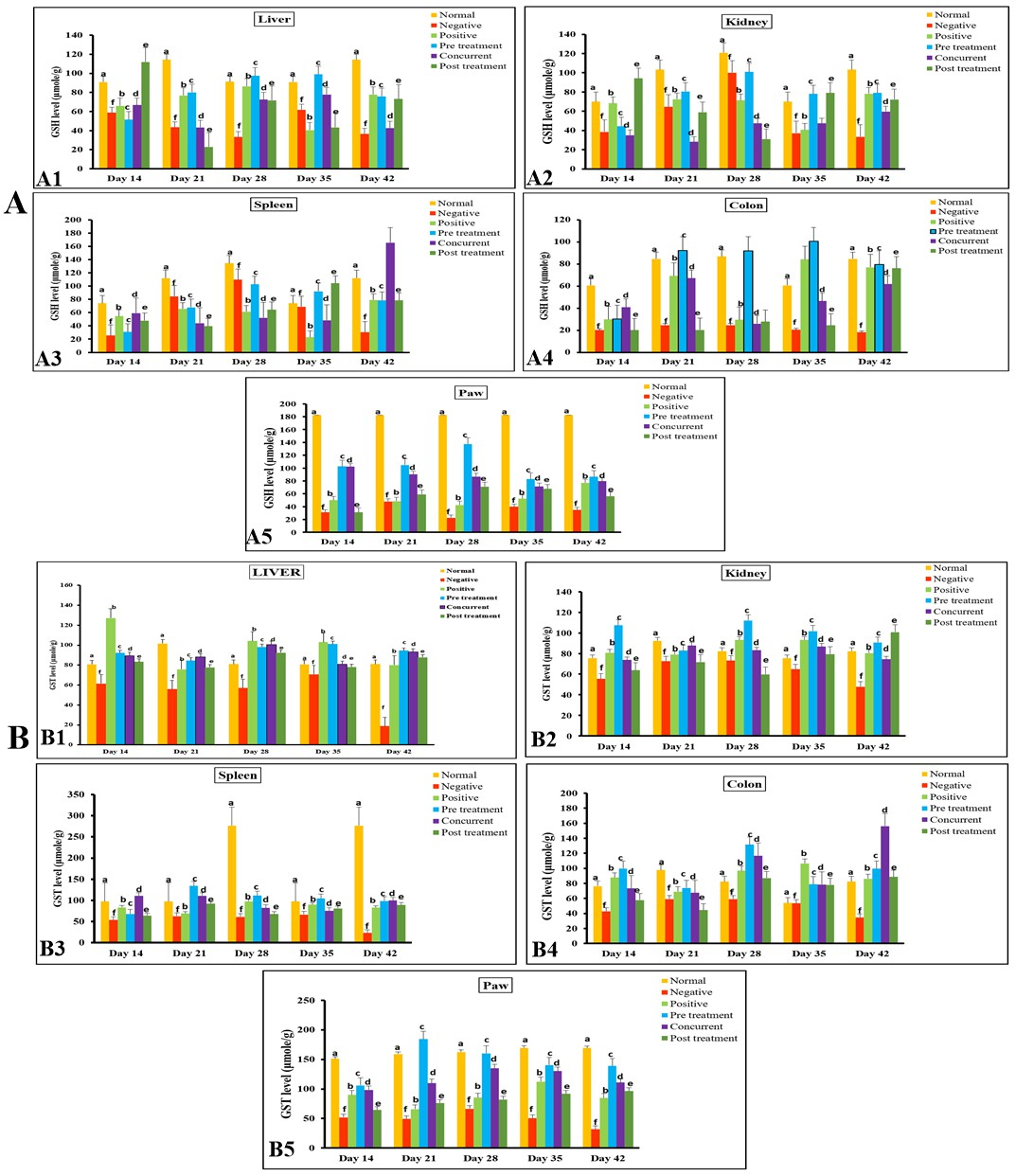

Supplement: S3 Fig — (A) Effect of P. acidilactici on GSH level in liver (A1), kidney (A2), spleen (A3), colon (A4), and paw (A5). (B) Effect of P. acidilactici on GST level in liver (B1), kidney (B2), spleen (B3), colon (B4), and paw (B5). Two way ANOVA followed by LSD for multiple comparisons (n = 5). The groups on the same day having different alphabetical superscripts are significantly different (p < 0.05). Normal – No treatment, Negative – CFA treatment, Positive – Dexamethasone treatment, Pre-treatment/Concurrent/Post-treatment – P. acidilactici. (TIF) [file pone.0324060.s003.tif]
